# Effectiveness of a transitional home care program in reducing acute hospital utilization: a quasi-experimental study

**DOI:** 10.1186/s12913-015-0750-2

**Published:** 2015-03-14

**Authors:** Lian Leng Low, Farhad Fakhrudin Vasanwala, Lee Beng Ng, Cynthia Chen, Kheng Hock Lee, Shu Yun Tan

**Affiliations:** Department of Family Medicine and Continuing Care, Academia, Level 4 Singapore General Hospital, Singapore, 169856 Singapore; Saw Swee Hock School of Public Health, National University of Singapore, National University Health System, Singapore, Singapore; Duke-NUS Graduate Medical School, Singapore, Singapore

**Keywords:** Transitional care, Home care, Healthcare utilization

## Abstract

**Background:**

Improving healthcare utilization is essential as health systems around the world grapple with the escalating demands for acute hospital resources. Evidence suggests that transitional care programs are effective to improve utilization of healthcare. However, the evidence for transitional care programs that enhance the home medical care model and provide multi-disciplinary patient-centered care is not well established. We evaluated if a transitional home care program operated by the Singapore General Hospital was effective in reducing acute hospital utilization.

**Methods:**

We performed a quasi-experimental study using a pre-post design to evaluate the effectiveness of a transitional home care program in reducing hospital admissions and emergency department attendances of medically complex patients enrolled into the program in a tertiary hospital in Singapore. Patients received a comprehensive needs assessment performed by the physician and a nurse case manager in the home setting, followed by an individualized care plan that included medical and nursing care, patient education and coordination of care with hospital specialists and community services. Primary study outcomes were emergency department attendances and hospital admissions to all hospitals. These were extracted from hospital administrative data and national health records. Wilcoxon Signed Ranks Test was used for assess differences in pre and post continuous data.

**Results:**

Overall, 262 patients were enrolled into the program and 259 were analyzed. Patients had a 51.6% and 52.8% reduction in hospital admissions in the three-month and six-month post enrollment, respectively. Similarly, a 47.1% and 48.2% reduction was observed for emergency department attendances in the three and six months post enrollment, respectively. The average difference in per patient hospital bed days in the pre- and post-enrollment periods were 12.05 days and 20.03 days at the 3-month and 6-month periods, respectively.

**Conclusions:**

Patients enrolled in the transitional home care program had significantly lower acute hospital utilization through the reduction of emergency department attendances and hospital admissions. A comprehensive assessment of patients’ medical and social needs in the home setting and formulation of an individualized care plan optimized post-discharge care for medically complex patients.

## Background

Health systems around the world are grappling with unprecedented challenges caused by rapid ageing of populations and increasing prevalence of chronic diseases. The rising demand for hospital resources is overwhelming all over the world - even for health systems of developed countries [1,2]. Singapore’s population is ageing four times more rapidly than other developed countries [2,3]. Perpetual high bed occupancies in hospitals [4] are placing tremendous pressure on acute care resources and hospitals to discharge patients to create beds for patients waiting in the emergency rooms. Singapore’s community and long term care sectors are less developed when compared to the acute care services. The lack of integration between acute care, long term care, and primary care is widely acknowledged. This gap further aggravates the problem resulting in a vicious cycle of discharge and readmissions to acute care hospitals [5,6].

Various transitional care programs aimed at reducing acute hospital utilization have been developed. Transitional care programs can be defined as a set of interventions, usually multi-disciplinary in nature, aimed at improving healthcare utilization through improving care coordination and continuity when patients transit between different healthcare settings.

There is evidence that transitional care programs are effective in reducing readmission rates, improving utilization of healthcare, and increasing patient satisfaction [7-10]. Examples of successful transitional care models include the advanced practice nurse led clinical program in the transitional care model (TCM) (developed by Mary Naylor) and the Care Transitions Intervention (CTI) (developed by Eric Coleman) [10,11].

Home medical care is commonly provided by primary care physicians or nurse practitioners as part of their comprehensive services for the purposes of promoting, maintaining or restoring health. However, the effectiveness of these home-based programs in reducing acute hospital utilization is not well established [12,13]. There are some transitional care programs that enhance the home medical care model and provide multi-disciplinary patient-centered care to patients discharged from hospital to home. Our objective was to evaluate the effectiveness of such a program that is operated by the Singapore General Hospital (SGH) in reducing acute hospital utilization. We hypothesized that patients who are enrolled into this program will have lower hospital readmissions and emergency department visits.

## Methods

### Study design

We performed a quasi-experimental study using a pre-post design on patients enrolled into the SGH transitional home care program (hereby referred to as the intervention program) between 1^st^ January 2008 and 31^st^ September 2012. Each patient served as his/her own control. The “pre” period was the 6 months before the intervention and the “post” period was the 6 months after enrollment into the intervention program. We did not use matched controls in the study design as there are important confounders affecting hospital utilization such as health literacy, social, and functional factors that could not be matched for when selecting historical controls from the hospital database [12,14].

The Institutional Review Board of Singapore Health Services approved the protocol, and waived the need for written informed consent from the participants.

### Study setting and population

SGH is the largest tertiary hospital in Singapore with 37 clinical specialties and 88,000 inpatient admissions each year [15]. Participants were adults admitted to SGH from 1^st^ January 2008 to 31^st^ September 2012 who were screened for suitability for enrollment into our program by their attending physicians in the hospital.

Inclusion criteria included patients with (a) multiple (at least 3) chronic medical co-morbidities requiring follow up, (b) Sub-acute medical conditions requiring follow up on discharge, and (c) who have limited mobility resulting in difficulty in accessing healthcare services. Sub-acute care was defined as patients with medical conditions that required close medical supervision after discharge from the hospital e.g. recent myocardial infarction or respiratory infection. Exclusion criteria were patients (a) who were independent in their activities of daily living, (b) without a caregiver at home, and (c) receiving other transitional care interventions.

### Intervention program (SGH transitional home care program)

A multi-disciplinary team comprising of a family physician, nurse case manager, physiotherapist, occupational therapist, speech therapist and medical social worker delivered the intervention. Nurses functioned as case managers. They also provided nursing care that included interventions such as nasogastric tube and urinary catheter insertions. They educated patients and their caregivers on self-management of chronic diseases such as congestive cardiac failure, chronic obstructive pulmonary disease, and diabetes mellitus.

The physician and nurse performed all first home visits together for a comprehensive assessment of the patient’s care needs within the first week of hospital discharge.

The interventions during the initial home visit assessment consisted of:

1. Optimizing medical conditions in the home setting
2. Educating patients and caregivers on self-management of chronic diseases by using action plans
3. Reducing polypharmacy and medication conflicts through medication reconciliation and facilitating adherence to treatment
4. Ensuring appropriate follow up by coordination of care with hospital specialists
5. Activating appropriate community services to support the patient’s care in the home setting

An individualized patient-centered care plan was drawn up for each patient after the initial home visit assessment. The frequency and timing of future home visits and the need to involve other members of the multi-disciplinary team was identified during the initial assessment and communicated to all team members involved in the care. Nurses made telephone call reviews based on patients’ care needs and were accessible to patients by phone during office hours. Early physician reviews were made based on the recommendations of the nurse case manager. The maximal duration of intervention was six months. Patients were transited out of the intervention program to a community primary care provider once their care needs have been stabilized to a manageable scale. The program was financed by fee for service, and the patients’ co-payment amount was determined by their financial status.

### Data collection, variables and study outcomes

Information was extracted from patients’ home care medical records, hospital administrative database, and the national electronic health records by the investigators. The medical records contained information on basic socio-demographics, medical co-morbidities, functional assessment, and detailed records of individual home care visits of all patients. Data extracted included socio-demographics such as age, gender, ethnicity, relationship of caregiver, admission ward class, medical co-morbidities, nursing needs, abbreviated mental test scores, modified barthel index, and discharge destination.

The primary study outcomes were hospital admissions, emergency department (ED) attendances, and length of stay at all hospitals in the three months pre- and post-enrollment. The secondary outcomes were hospital admissions and emergency department (ED) attendances and hospital length of stay at all hospitals in the six months pre- and post-enrollment. These were extracted from hospital administrative data and national health records. The national health records captured admissions and ED attendances to all hospitals in Singapore.

### Statistical analysis

Wilcoxon Signed Ranks Test was used for assess differences in pre and post continuous data. Statistical significance was set at the conventional P <0.05. Sensitivity analyses were performed excluding patients with only one home care visit and patients with deaths within 6 months of enrollment. All statistical analyses were performed using SPSS Version 17.0 (IBM Corp, NY, USA).

## Results

Overall, 262 patients were enrolled into the program and 259 were analyzed. Three (0.011%) patients were excluded from analysis because of incomplete data.

Table 1 shows the socio-demographics, clinical profile, and outcomes of the patients. Patients were mostly elderly (86.9% aged 65 years and above). Majority were from admission ward class B2 and C (an indicator of lower socio-economic status) and dependent in their functional status with low Barthel scores and requiring high nursing care needs e.g. nasogastric feeding tubes, pressure ulcers and indwelling urinary catheters. The top five medical comorbidities were cerebrovascular accident, diabetes mellitus, pneumonia, dementia and heart failure.

**Table 1:** **Socio-demographics, clinical profile and outcomes of patients enrolled into SGH transitional home care program**

	**n (%)**
**Total no. of patients**	**259**
**Age group**	
<65 years	34 (13.1)
≥65 years	225 (86.9)
**Gender**	
Male	104 (40.2)
Female	155 (59.8)
**Ethnicity**	
Chinese	200 (77.2)
Malay	33 (12.7)
Indian	21 (8.1)
Others	5 (1.9)
**Marital status**	
Single	13 (5)
Married	188 (72.6)
Widowed	48 (18.5)
Separated/divorced	5 (1.9)
**Admission ward class**	
A	12 (4.6)
B1/B2+	13 (5)
B2	95 (36.7)
C	103 (39.8)
**Co-morbidities**	
Stroke	135 (52.1)
Diabetes mellitus	111 (42.9)
Pneumonia	91 (35.1)
Dementia	68 (26.3)
Heart failure	57 (22)
Chronic kidney disease	51 (19.7)
Depression	33 (12.7)
Cancer	32 (12.4)
Gout	20 (7.7)
COPD	20 (7.7)
**Nursing needs**	
Nasogastric tube	148 (57.1)
Pressure ulcers	135 (52.1)
Indwelling urinary catheter	53 (20.5)
Tracheostomy tube	16 (6.2)
**Abbreviated mental status score**	
<7	190 (73.5)
≥7	41 (15.8)
**Barthel score**	
<10	231 (89.2)
≥10	28 (10.8)
**Discharge destination (s)**	291
Specialist outpatient clinic	77 (26.5)
General practitioner/polyclinic	67 (23.0)
Home Medical service	61 (21.0)
Home Hospice service	43 (14.8)
Home Nursing service	37 (12.7)
Nursing Home	6 (2.1)
**Six-month mortality rate**	41 (15.8)
**Number of home visits**	
1	71 (27.4)
2	35 (13.5)
3	31 (12.0)
4	39 (15.1)
5	29 (11.2)
6	22 (8.5)
7+	30 (11.6)

Majority of patients (59.1%) had at least three home visits during the intervention period. About 26.5% of our patients had to be transited to the specialist outpatient clinics on discharge from our program because the care complexity was beyond the capabilities or capacities of available community primary care providers. Twenty three (23%) of the patients were transited to long term home medical care providers in the community. The 6-month mortality rate of patients enrolled in the program was 15.8% or 41 total deaths (Table 1). Patients who demised were included in all the statistical analysis.

**Figure 1:**
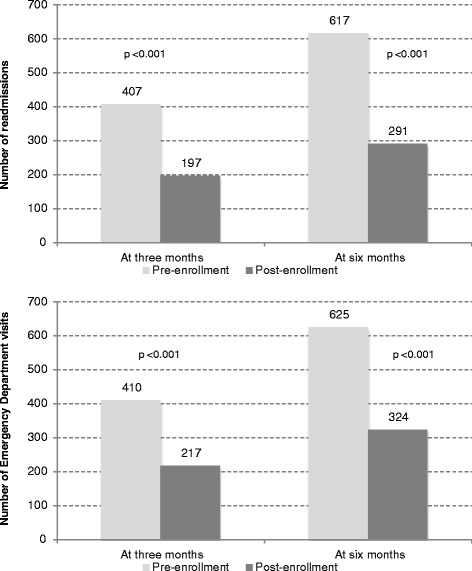
**Number of admissions and emergency department visits at three and six month stratified by pre and post enrollment.**

The total number of hospital admissions three months prior to enrollment was 407. The total number of hospital admissions in the three months post enrollment was 197 demonstrating a reduction of 51.6% (p < 0.001) (Figure 1, Table 2). This effect was also seen at the total hospital admissions at the 6 month period; 617 pre-enrollment admissions vs. 291 post-enrollment admissions - a 52.8% reduction (p < 0.001) (Table 2).

**Table 2:** **Pre- and Post-enrollment acute hospital utilization, length of stay and specialist outpatient clinic visits at 3 and 6 month**

	**Pre-enrollment**	**Post-enrollment**	**Difference (pre - post)** ^**a**^	**P value** ^**b**^
**Admissions**				
3-months from intervention	407	197	210	<0.001
6-months from intervention	617	291	326	<0.001
**Emergency department attendances**				
3-months from intervention	410	217	193	<0.001
6-months from intervention	625	324	301	<0.001
**Length of stay (days)**				
3-months from intervention	5611	2490	3121	<0.001
6-months from intervention	9463	3676	5187	<0.001
**Specialist outpatient clinic visits**				
3-months from intervention	312	421	−109	0.004
6-months from intervention	617	673	−56	0.869

^a^-ve: post higher utilization.

^b^Wilcoxon sign rank test.

The total numbers of ED attendances three months prior to enrollment was 410 compared to 217 visits in the post-enrollment period. The observed reduction was 47.1% (p < 0.001) (Figure 1, Table 2). The reduction of ED attendances was sustained even at the 6 month period; 625 pre-enrollment attendances vs. 324 post-enrollment attendances representing a 48.2% reduction (p < 0.001) (Figure 1, Table 2). The average difference in per patient hospital bed days in the pre- and post-enrollment periods were 12.05 days and 20.03 days at the 3-month and 6-month periods, respectively. In other words, patients who were enrolled into the program on the average require much shorter hospital stays in the post-enrollment period.

There was a significant increase in the specialist outpatient clinic (SOC) visits between pre- and post-enrollment at 3 months (p = 0.004). However at the 6 month period, the increase was not statistically significant (p 0.869) (Table 2). After sensitivity analyses were performed excluding patients with a shorter duration of intervention (such as patients with only one home care visit or patients with deaths within 6 months of enrollment), the reduction in admissions, ED attendances and length of stay remained statistically significant (Table 3).

**Table 3:** **Pre- and Post-enrollment acute hospital utilization, length of stay and specialist outpatient clinic visits at 3 and 6 months**

	**Pre-enrollment**	**Post-enrollment**	**Difference (pre - post)** ^**a**^	**P value** ^**b**^
**Exclude one homecare visit only (n = 186)**				
**Re-admission visits**				
3-months from intervention	298	152	146	<0.001
6-months from intervention	463	226	237	<0.001
**Emergency department attendances**				
3-months from intervention	290	176	114	<0.001
6-months from intervention	451	262	189	<0.001
**Length of stay (days)**				
3-months from intervention	3981	1572	2409	<0.001
6-months from intervention	6675	2543	4132	<0.001
**Specialist outpatient visits**				
3-months from intervention	232	340	−108	0.003
6-months from intervention	474	559	−85	0.479
**Exclude deaths within 6 months of intervention (n = 218)**				
**Re-admission visits**				
3-months from intervention	351	151	200	<0.001
6-months from intervention	525	234	291	<0.001
**Emergency department visits**				
3-months from intervention	355	173	182	<0.001
6-months from intervention	535	266	269	<0.001
**Total length of stay (days)**				
3-months from intervention	4627	1973	2654	<0.001
6-months from intervention	7599	3016	4583	<0.001
**Specialist outpatient visits**				
3-months from intervention	268	397	−129	<0.001
6-months from intervention	533	645	−112	0.177

^a^-ve: post higher utilization.

^b^Wilcoxon sign rank test.

## Discussion

Our study showed that a multi-disciplinary transitional home care program in Singapore was effective in reducing acute hospital utilization for patients with multiple medical co-morbidities in the three and six months after enrollment. The patients had significant reduction in hospital admissions, ED attendances and hospital length of stay in the three-month and six-month periods after enrollment into the program and supported both our hypotheses for the primary and secondary outcomes. The greatest benefit was in the first three months of intervention and coincided with the period where transitional care was most intensive. Although actual expenditures were not obtained in this evaluation, we estimated the savings to the public health system from the difference in hospital stays, emergency department attendances saved against the additional costs of home visits and specialist outpatient clinic visits. The cost of a combined doctor and nurse home visit during the program, SOC visit (repeat consultation by consultant at private rates), emergency department visit and average bed cost per patient day (excluding government subsidies) were S$261, S$75, S$216 and S$842 respectively [5,16]. The total costs of home visits and additional SOC visits were S$229, 997 and S$4,200 respectively. There were 5,787 bed days saved with an associated cost savings of S$4.87 million and 301 fewer emergency department attendances with associated cost savings of S$65,016. Therefore the overall cost savings from reductions in admissions and emergency department attendances attributed to our program for 259 patients was estimated to be S$4.7 million. This crude estimate assumes no additional healthcare expenditure by patients on the program and that the costs of home visits fully remunerated for the doctor and nurse time spent. This assumption is reasonable given that patients on the program did not receive other transitional care programs, and expenditure on medications, equipment and community services are likely to be incurred by patients with similar care needs in other transitional care programs. The doctor and nurse in the program assumed other responsibilities in the department that accounted for their time when they were not doing home visits. Notwithstanding patients deaths that resulted in fewer readmissions in the post-enrollment period and an in-depth cost-effectiveness analysis, our program appeared to provide cost savings for patients and the health system.

This program targets patients that are among the sickest in the system as evidenced by the age, co-morbidities, and level of functional impairment of the participants. Such patients are known to be high utilizers of hospital resources. A comprehensive assessment of the patient’s medical and social care needs and the formulation of an individualized care plan optimized the care in the home setting. Considering the challenges of managing such patients without hospital resources and the complexity of the care needed, the good outcomes achieved are encouraging and supports the implementation of more such programs for patients with complex care needs.

The favorable results of our program were consistent with a home based palliative care program [17] and shared similarities in patient characteristics such as home bound status, dependency in functional status and high nursing care needs; and interventions that are multi-disciplinary in nature and accessibility to a nurse by phone. A home-based intervention program for heart failure patients consisting of a single home visit within one week of discharge by a nurse and pharmacist to optimize medication management and identify early clinical deterioration [18] and a physician-directed transitional care program that provided early review at home or clinic setting within 72 hours of discharge, dietary education and follow-up phone reviews reported reductions in unplanned readmissions [19]. Similar to our program, these two programs targeted patients with high disease burden and baseline readmission risk; and emphasized on early review, medication reconciliation and patient education in the post-discharge period. Although the significant heterogeneity in intervention content and context limited meaningful comparisons between studies to identify a single intervention or bundle of interventions that reliably reduced readmissions [20], the results from these studies and our program add to the increasing evidence that supports the effectiveness of multi-disciplinary teams, and the importance of optimizing medical care, being accessible to patients in times of crisis and early review in the post-discharge period to reduce utilization of acute hospital care. A review by Verhaegh et al. suggested that to reduce readmissions, transitional care interventions should consist of high intensity interventions that include care coordination by a nurse, communication between the primary care provider and hospital and a home visit within three days of discharge [21]; which were core components of our program.

The increase in SOC visits post-enrollment was not surprising. It could be attributed to the discovery of unresolved clinical care issues that require specialist input and the transition to SOC of some patients with complex care needs that was beyond the capabilities of community primary care providers.

Family Medicine training of the physician may have contributed to the effectiveness of this transitional care program. As a specialty, Family Medicine emphasizes on providing care that is primary, personal, preventive, comprehensive, continuing, and community oriented. This care paradigm is highly compatible with the objectives of transitional care programs. Caring for patients at home is also distinctive of family medicine. Appropriate use of time and resources as well as coordinating care with specialists and multidisciplinary team members are major competencies required of family physicians. These characteristics of family medicine are favorable and should be capitalized in home-based transitional care programs.

Notwithstanding the encouraging results of this study, there are important limitations to consider. Firstly, the most important is the limitation of the pre-post design in which patients were used as their own controls. There is the possibility that the patients’ acute hospital utilization will decline naturally without intervention. However, the patients were medically complex and the progressive nature of their illnesses makes regression to the mean relatively unlikely. A randomized controlled trial would have provided a higher level of evidence for evaluation of effectiveness. However, the promising results from this retrospective study have supported the re-design of a new 3-month transitional home care service in the hospital. Secondly, our hospital database and medical records did not allow us to account for out-of hospital deaths or migration if patients have exited our program before 6 months. However, we believe these numbers are likely to be small as almost all patients were followed up for at least three months to meet our primary outcome measure. Moreover, elderly people in Singapore die mainly in hospitals [22] and it is unlikely that we would have missed this data. The functional dependence of majority of our patients also made them unlikely to move out of the country. In addition, we have performed sensitivity analysis by excluding patients who died before six months and analyzed at three and six months post-enrollment. Thirdly, the results of our study are based on home and bed-bound patients discharged from an acute hospital in Singapore and may have limited generalizability beyond this context and setting. Finally, while there are reasons to believe that this transitional care program provided cost savings for patients and the health system, we did not have full data on our program’s startup costs, implementation costs and additional costs to the patients to evaluate the exact cost-effectiveness of our program.

## Conclusions

Patients enrolled in the SGH transitional home care program had significantly lower rates of hospital utilization through the reduction of hospital admissions and emergency department attendances. This benefit is the greatest in the first three months of enrollment. Our findings add to the increasing evidence that supports the effectiveness of a multi-disciplinary transitional care programs in reducing utilization of hospital resources. A multidisciplinary approach based on the paradigm of family medicine may have contributed to the effectiveness of this transitional care program. Future transitional care programs using a randomized control trial design and measuring other clinical outcomes such as quality of life and cost-effective analyses are needed to provide more precise information of the clinical and economic impact of such programs.

## Abbreviations

SGH: Singapore general hospital
ED: Emergency department
SOC: Specialist outpatient clinic
